# Neuroendocrine Differentiation of Prostate Cancer Metastases Evidenced “in Vivo” by ^68^Ga-DOTANOC PET/CT: Two Cases

**DOI:** 10.14740/wjon739w

**Published:** 2014-05-06

**Authors:** Giordano Savelli, Alfredo Muni, Roberto Barbieri, Giuseppe Valmadre, Giorgio Biasiotto, Chiara Minari, Claudio Ghimenton, Renato Pagani, Elisa Pecini, Matteo Falcone

**Affiliations:** a Nuclear Medicine Unit, “Carlo Poma” Civic Hospital, Strada Lago Paiolo, 10 - 46100 Mantua, Italy; b Nuclear Medicine Unit, SS. Antonio e Biagio e Cesare Arrigo Hospital, 46100 Alessandria, Italy; c Nuclear Medicine Unit, Biochemical Section, University of Brescia, 25125 Brescia, Italy

**Keywords:** Positron emission tomography, Somatostatin receptors, Castration-resistant prostate cancer

## Abstract

Prostate cancer is the second most commonly diagnosed neoplasm in men. This neoplasm has usually excellent prognosis, mostly consequent to the early diagnosis and the effective hormonal therapy. However, significant percentages of patients treated with total androgen blockade therapy, escape to treatment and evolve toward a more aggressive type of cancer. This clinical entity, named castration-resistant prostate cancer, has few and less effective therapeutic opportunities. Therefore, any additional information concerning possible biological targets to therapy is welcome. Here we describe two cases in which ^68^Ga-DOTANOC PET/CT evidenced the somatostatin receptor overexpression by prostate metastases. The presence of these receptors may support with a more strong evidence the possibility to administer somatostatin analogs as an adjuvant therapy.

## Introduction

The possibility of neuroendocrine differentiation (NED) of prostate cancer (PC) is a consolidate statement. Some researchers have used chromogranin A as serological maker of neuroendocriny and, therefore, to select patients affected by metastatic PC to be treated with somatostatin analogs. Unfortunately, chromogranin A is a highly non-specific maker, thus its elevation does not imply the overexpression of somatostatin receptors (SSTRs). Aim of an ongoing phase III trial with the positron emitter radiopharmaceutical [^68^Ga-DOTA,1-Nal3]octreotide positron emission tomography (PET) at our hospital is to detect “*in vivo*” the overexpression of SSTRs in patients with metastatic PC. The phase III trial (EUDRA CT number 2010-021026-35) granted by the RegioneLombardia “Call for independent research 2009” was aimed to detect SSTRs overexpression in skeletal and soft tissue metastases of patients affected by castration-resistant prostate cancer (CRPC). [^68^Ga-DOTA,1-Nal3]octreotide was synthesized on site following the procedure reported in the literature. PET/CT scan was carried out with a Siemens Biograph 6 PET/CT scanner, 1 h after the intra-venous administration of the 120 - 140 MBq of the radiopharmaceutical. The acquisition parameters for the CT scan were: kV = 130; effective mAs = 70; maximum reconstructed width = 5 mm without overlap; pitch 1.5 mm; standard reconstruction algorithm. PET scan was performed from the lower thighs, with 6 bed positions (3 min per bed). PET was reconstructed using standard algorithms provided by Siemens. Eight patients meeting the recruitment criteria were studied in the first 6 months. Among these, [^68^Ga-DOTA,1-Nal3]octreotide PET/CT evidenced SSTRs overexpression in skeletal and lung metastases of two patients. [^68^Ga-DOTA,1-Nal3]octreotide PET/CT may detect NED of CRPC. This finding could warrant the patients more targeted therapies.

## Cases Report

### Case 1

In April 2011, MC underwent a transurethral radical prostatectomy for benign prostatic hyperplasia with the unexpected discovery of one adenocarcinoma GS (4+4). Castration therapy with triptorelin every 3 months and bicalutamide 50 mg/daily was scheduled. In June 2011, a bone scan detected multiple bone metastases. In July 2012, a CT scan depicted progressive disease including lung metastases. Patient refused chemotherapy, thus zoledronic acid 4 mg every 3 weeks started. In September 2012, the patient started oxycodone therapy. Due to the patient’s refusal of chemotherapy, the oncologist decided to study SSTRs expression of the neoplasm with the aim of treating him with somatostatin analogs.

### Imaging findings

**Figure 1:**
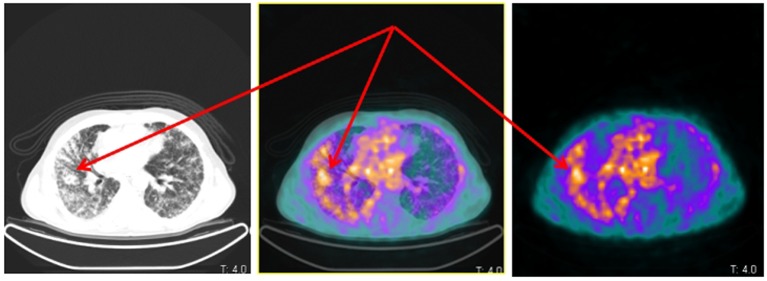
^68^Ga-DOTANOC transaxial PET/CT (left CT attenuation correction, middle fused PET/CT, right PET only) showing increased uptake of the radiopharmaceutical in the left sovra-acetabular region corresponding to a mixed (predominantly lytic) skeletal metastasis (arrows).

**Figure 2:**
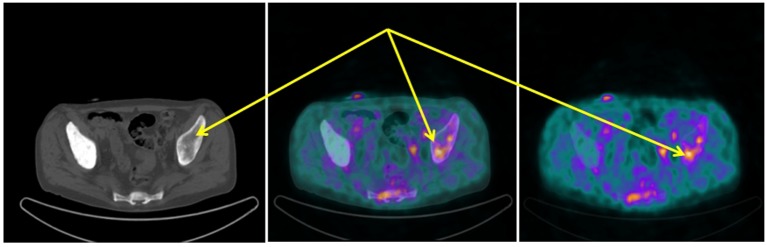
Same patient and acquisition parameters of Fig. 1. ^68^Ga-DOTANOC transaxial PET/CT (left CT attenuation correction, middle fused PET/CT, right PET only) showing increased uptake corresponding to lymphangitic carcinomatosis of the right lung (arrows).

[^68^Ga-DOTA,1-Nal3]octreotide (^68^Ga-DOTANOC) PET/CT showed several areas of increased uptake located in bone metastases. The uptake was more significant in metastases with a lytic component on CT (Fig. 1). Moreover, two diffuse areas of increased lung uptake corresponding to lymphangitic neoplastic spreads were noted (Fig. 2). These finding were consistent for an NED of neoplastic cells with overexpression of SSTR2, SSTR3 and SSTR5.

### Case 2

In July 2013, RS, a 55-year-old male, was admitted to a local hospital because of temporal and occipital headache, left sub-mandibular swelling and left tongue deviation. Rachicentesis, fibroscopic assessment, ultrasound, head and neck contrast enhanced CT and MRI resulted negative. PSA reported 407 ng/mL (n.v. < 4 ng/mL). The CT scan evidenced enlarged prostate gland, infiltration of the bladder wall, right external iliac nodes and multiple skeletal osteoblastic metastases (confirmed by a bone scan). Bicalutamide 50 mg/day and leuprolide 3.75 mg/28 dd were started. The prostate biopsy evidenced the coexistence of adenocarcinoma (GS 4+4) and neuroendocrine (NE) cells in the entire eight specimen obtained. The final diagnosis was, therefore, prostate adenocarcinoma with a very high NE component.

### Imaging findings

**Figure 3:**
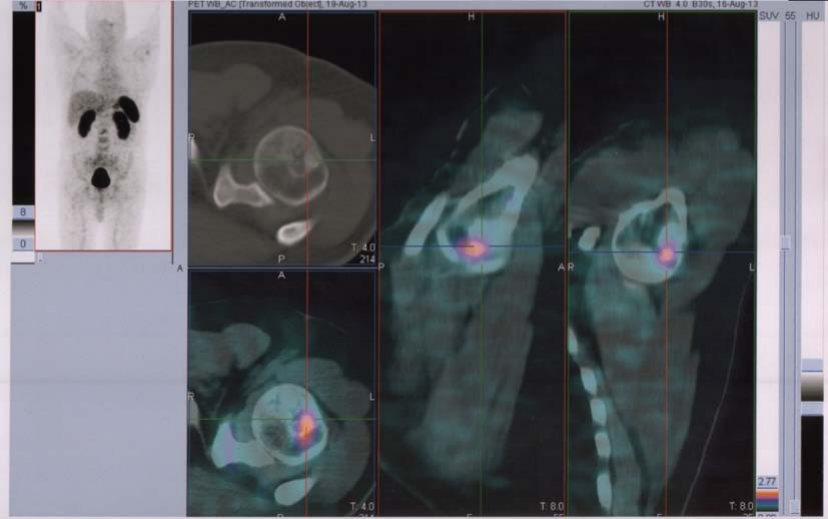
^68^Ga-DOTANOC transaxial PET/CT at the level of left humeral head showing increased uptake of the radiopharmaceutical in a mixed metastasis (pointers).

**Figure 4:**
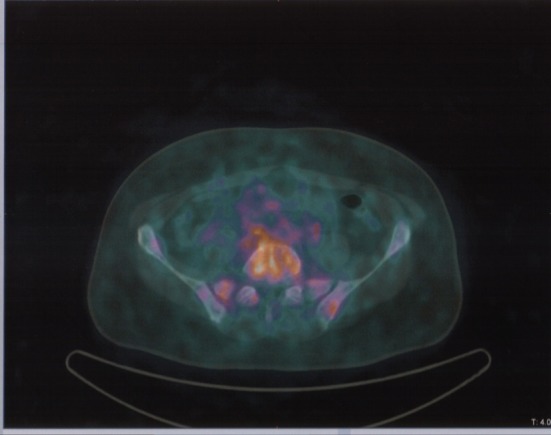
Same patient and acquisition parameters of Fig. 3. ^68^Ga-DOTANOC transaxial PET/CT at the level of fourth lumbar vertebra showing increased uptake of the radiopharmaceutical in a mixed metastasis.

In August 2013, the patient referred to our hospital to investigate “*in vivo*” SSTR receptorial status. PET/CT evidenced several areas of increased uptake scattered through the whole skeleton, corresponding on CT with mostly mixed osteoblastic/osteolytic metastases (Fig. 3, 4).

## Discussion

Standard first-line treatments for CRPC are docetaxel plus prednisone but the outcome is remarkably low. In a lesser degree, the same remark may be drawn for newer second option treatment such cabazitaxel, abiraterone acetate and ezalutamide which have added nearly 3 months of survival. These simple summaries should be sufficient to call for newer and more effective treatments.

The prostate epithelium is composed of basal, luminal secretory and NE cells. The first two types may give rise to adenocarcinomas, whereas NE cells generate NE neoplasm. Indeed, “NED” in PC generally encompasses NE carcinoma (such as small cell carcinoma), as well as prostatic adenocarcinoma with focal NED. Some studies suggested that NED indicates an adverse prognosis [1] whereas others correlate it to a treatment response [2]. The knowledge of mechanisms inducing NED in PC is scant, albeit androgen withdrawal is thought to be a promoting factor [3]. Serum chromogranine A (CgA) test has been the main parameter to detect NED in order to treat the patients with somatostatin analogs. However, CgA suffers of a low specificity [4-6]. There may be several causes of CgA elevation that are unrelated to the onset of neoplasms. CgA elevation is present with various gastro-intestinal, cardiovascular, pulmonary, rheumatologic, renal and endocrine diseases such as chronic atrophic gastritis, liver cirrhosis, chronic hepatitis, pancreatitis, inflammatory bowel disease, *Helicobacter pylori* infection and irritable bowel syndrome. Furthermore, NED in neoplastic diseases is not synonymous of SSTR expression [7]. In summary, CgA is a surrogate marker and should not be used as the evidence of SSTRs overexpression. Thus, a reliable and easy to use method to study SSTR presence is mandatory.

Nuclear medicine procedures with gamma emitting radiopharmaceuticals have been occasionally employed in the past to detect SSTR overexpression in PC [8-10]. However, gamma camera technology has an intrinsic spatial resolution which limits its use. In the last few years, they become available some somatostatin analogs PET radiopharmaceuticals (^68^Ga-DOTATOC, ^68^Ga-DOTANOC). ^68^Ga-DOTANOC was originally developed to study NE carcinomas and in this setting it has shown to have better figures of merits for the study of unknown primary tumors, lymph nodes and skeletal metastases. However, SSTRs are widely distributed in various neoplasm; therefore, ^68^Ga-DOTANOC may be potentially used in different clinical settings.

The first report with ^68^Ga-DOTATOC in PCs revealed a weak uptake of metastases and suggested the use of different compounds [11].

Our phase III trial contemplates the use of ^68^Ga-DOTANOC which differs from ^68^Ga-DOTATOC in one amino acid in position 3 of octreotide and, thus, has different SSTR affinities (DOTANOC binds to subtype 2, 3 and 5, whereas DOTATOC to subtypes 2 and 5).

The first of our cases evidences ^68^Ga-DOTANOC uptake in skeletal metastases and in lung lymphangitic carcinomatosis. This is an intriguing finding, since there are some evidences suggesting a different modality of differentiation between skeletal and soft tissue metastases in PC. Moreover, skeletal metastases evidenced a mixed pattern of ^68^Ga-DOTANOC uptake within the lesions with lytic metastases somehow taking up more radiopharmaceutical than blastic ones.

The second case showed a high uptake in bone metastases, mainly in the lytic component as well. The first, more direct, and easier explanation of this behavior is that the dense, compact bone, forming the blastic metastases, has reduced cellularity, fewer SSTRs, and therefore less uptake. A second, more intriguing, not alternative, explanation reading is that lytic bone metastases usually represent a “late” form of bone metastases. Indeed, PC skeletal metastases evolve from an early and more differentiated type after onset to a late less differentiated type [12-14]. This second group of metastases could harbor cells with NED.

Which is the added value of ^68^Ga-DOTANOC examination in PC patients? Basically, the *in vivo* detection of NED brings with it the option related to somatostatin analogs therapy, both in the naive or radiolabeled form. ^68^Ga-DOTANOC PET/CT may evidence if the patient was affected by a PC with NED, if NED has evolved to produce SSTR and if it is reasonable to administer the patient with somatostatin analogs. The pharmacogenomic era promises to advance towards an always more personalized therapy. Thus, the attempt carried out in the past to treat CRCP patients with somatostatin analogs, without effectively to know the SSTRs status, looks like a shot in the dark which can be justified only by the lack of valid solutions. We suggest that trials aiming to evaluate SSTR mediated treatments in PC will recruit patients after the *in vivo* visualization of SSTR.

## References

[1] Cohen MK, Arber DA, Coffield KS, Keegan GT, McClintock J, Speights VO (1994). Neuroendocrine differentiation in prostatic adenocarcinoma and its relationship to tumor progression. Cancer.

[2] Deng X, Elzey BD, Poulson JM, Morrison WB, Ko SC, Hahn NM, Ratliff TL (2011). Ionizing radiation induces neuroendocrine differentiation of prostate cancer cells in vitro, in vivo and in prostate cancer patients. Am J Cancer Res.

[3] Marchiani S, Tamburrino L, Nesi G, Paglierani M, Gelmini S, Orlando C, Maggi M (2010). Androgen-responsive and -unresponsive prostate cancer cell lines respond differently to stimuli inducing neuroendocrine differentiation. Int J Androl.

[4] Campana D, Nori F, Piscitelli L, Morselli-Labate AM, Pezzilli R, Corinaldesi R, Tomassetti P (2007). Chromogranin A: is it a useful marker of neuroendocrine tumors?. J Clin Oncol.

[5] Lawrence B, Gustafsson BI, Kidd M, Pavel M, Svejda B, Modlin IM (2011). The clinical relevance of chromogranin A as a biomarker for gastroenteropancreatic neuroendocrine tumors. Endocrinol Metab Clin North Am.

[6] Bech PR, Ramachandran R, Dhillo WS, Martin NM, Bloom SR (2012). Quantifying the effects of renal impairment on plasma concentrations of the neuroendocrine neoplasia biomarkers chromogranin A, chromogranin B, and cocaine- and amphetamine-regulated transcript. Clin Chem.

[7] Matei DV, Renne G, Pimentel M, Sandri MT, Zorzino L, Botteri E, De Cicco C (2012). Neuroendocrine differentiation in castration-resistant prostate cancer: a systematic diagnostic attempt. Clin Genitourin Cancer.

[8] Nilsson S, Reubi JC, Kalkner KM, Laissue JA, Horisberger U, Olerud C, Westlin JE (1995). Metastatic hormone-refractory prostatic adenocarcinoma expresses somatostatin receptors and is visualized in vivo by [111In]-labeled DTPA-D-[Phe1]-octreotide scintigraphy. Cancer Res.

[9] Thakur ML, Kolan H, Li J, Wiaderkiewicz R, Pallela VR, Duggaraju R, Schally AV (1997). Radiolabeled somatostatin analogs in prostate cancer. Nucl Med Biol.

[10] Kalkner KM, Nilsson S, Westlin JE (1998). [111In-DTPA-D-Phe1]-octreotide scintigraphy in patients with hormone-refractory prostatic adenocarcinoma can predict therapy outcome with octreotide treatment: a pilot study. Anticancer Res.

[11] Luboldt W, Zophel K, Wunderlich G, Abramyuk A, Luboldt HJ, Kotzerke J (2010). Visualization of somatostatin receptors in prostate cancer and its bone metastases with Ga-68-DOTATOC PET/CT. Mol Imaging Biol.

[12] Morrissey C, Lai JS, Brown LG, Wang YC, Roudier MP, Coleman IM, Gulati R (2010). The expression of osteoclastogenesis-associated factors and osteoblast response to osteolytic prostate cancer cells. Prostate.

[13] Roudier MP, Vesselle H, True LD, Higano CS, Ott SM, King SH, Vessella RL (2003). Bone histology at autopsy and matched bone scintigraphy findings in patients with hormone refractory prostate cancer: the effect of bisphosphonate therapy on bone scintigraphy results. Clin Exp Metastasis.

[14] Lee Y, Schwarz E, Davies M, Jo M, Gates J, Wu J, Zhang X (2003). Differences in the cytokine profiles associated with prostate cancer cell induced osteoblastic and osteolytic lesions in bone. J Orthop Res.

